# Selected conservation management strategies enhance maize yield stability in the sub-humid tropical agro-ecozone of Upper Eastern Kenya

**DOI:** 10.1038/s41598-023-49198-8

**Published:** 2023-12-08

**Authors:** Milka Kiboi, Collins Musafiri, Andreas Fliessbach, Onesmus Ng’etich, Isaiah Wakindiki, Felix Ngetich

**Affiliations:** 1 Department of International Cooperation, Research Institute of Organic Agriculture (FiBL), Ackerstrasse 113, 5070 Frick, Switzerland; 2 Cortile Scientific Limited, PO Box 34991-00100, Nairobi, Kenya; 3 Department of Soil Science, Research Institute of Organic Agriculture, (FiBL), Ackerstrasse 113, 5070 Frick, Switzerland; 4 Department of Water and Agricultural Resource Management, University of Embu, PO Box 6, 60100 Embu, Kenya; 5 KCA University, PO Box 56808-00200, Nairobi, Kenya; 6 Research Centre for Smallholder Farmers, PO Box 10451, 30100 Eldoret, Kenya; 7 School of Agricultural and Food Sciences, Jaramogi Oginga Odinga University of Science and Technology (JOOUST), PO Box 210-40601, Bondo, Kenya

**Keywords:** Agroecology, Climate change

## Abstract

Conservation management strategies have been recommended to enhance soil fertility, moisture retention, crop yield, and yield stability in rainfed agriculture. However, there is limited research on yield stability. We evaluated the effect of integrating soil inputs in conservation tillage on yield and yield stability in Meru South, Upper Eastern Kenya, for eleven consecutive cropping seasons. The trial treatments included conservation tillage without soil inputs (Mt), conservation tillage with soil inputs: sole inorganic fertilizer (F), residue + inorganic fertilizer (RF), residue + inorganic fertilizer + manure (RFM), residue + manure + legume *Dolichos Lablab* L. (RML), residue + Tithonia + manure (RTM), residue + Tithonia + phosphate rock (RTP) and conventional tillage (Control). Conservation tillage with RFM was the best-fit strategy for enhancing yields. There was heterogeneity in yield residual variance. A larger residual variance implied lesser yield stability. Mt treatment had the least yield residual variance of 0.12 Mg ha^−2^, followed by Ct and RML, 0.15 Mg ha^−2^, while RTM had the highest yield residual variance of 0.62 Mg ha^−2^. Contrarily, the most stable treatments had the least average yields. The study indicated a positive influence of incorporating soil inputs in conservation tillage on yield and suggests longer-term research for yield stability.

## Introduction

Increasing farmer yields and returns of staple cereal crops such as maize, wheat, and rice in developing countries has been a major goal of agricultural development strategies since the Green Revolution^1^. However, this has not been achieved in sub-Saharan Africa (SSA) countries, Kenya included^2^, where maize is grown by most households on rain-fed agricultural land. Low macro-nutrient levels, continuous crop cultivation without incorporating soil inputs, increasing population, and climate variability are among the major impediments to agricultural growth in SSA and our study area, Kenya^3^. Kenya is one of the hydroclimatic regions subject to extreme rainfall variability, water scarcity, droughts, and floods^4^. Increasing crop productivity to meet the growing human population's rising food demands calls for implementing climate-resilient and sustainable agricultural strategies such as conservation agriculture (CA) or conservation management strategies^5^.

Conservation management strategies are defined as practices that (1) enhance soil conservation and water-holding capacity, (2) increase crop yield, and (3) yield stability under the smallholder rainfed farming system^6^. The strategies could include any of the three principles of conservation agriculture: (1) reduced tillage, (2) maintenance of soil cover, and (3) crop rotation/intercropping^7^. Conservation management strategies have been extensively promoted worldwide and in SSA as a pathway to combat soil nutrient depletion and moisture stress, land degradation, and increase crop productivity^8^. Conservation agriculture has been recommended as a sustainable substitute for conventional maize production practices^9^ under rainfed conditions^10^. Yet, the majority of the farmers in SSA continuously practice conventional agricultural practices. Conventional agriculture involves constant soil disturbance and crop residue removal, which have been linked to soil degradation by causing soil erosion and compaction, reducing nutrient and water-holding capacities, and destroying habitats for beneficial soil organisms^11^. Several studies have reported increased yields under CA compared to the conventional system^12,13^. However, others report no differences or decreases between CA and conventional strategies^10^. After conducting research for four consecutive cropping seasons in the study area, Kiboi et al.^14^ reported no significant difference in yields between conservation and conventional tillage systems. This could be attributed to the long period required for yield increment under conservation tillage^15^. Thus, there is a need to incorporate soil fertility inputs and assess their effect on crop productivity over a longer term under conservation strategies.

Soil inputs are critical in soil fertility management and crop productivity. Sole inorganic fertilizer use has been observed to increase yields^16^. However, farmers apply them in insufficient quantities due to their high costs in the study area and unavailability^17^. Most smallholder farms' locally available organic inputs are limited in quantity and quality^14^. Integrating organic or inorganic inputs has been suggested as the most promising management strategy for increasing crop yields^18^. Cai et al.^19^ found that combining manure with synthetic fertilizer significantly increased maize crop yields. Further, integration of the inputs with conservation strategies improves crop productivity even in the short term^20^. For example, research conducted in the study area by Mutuku et al.^21^ found that conservation tillage with residue retention and manure significantly increased maize grain yield within two years (four cropping seasons). Kaupa and Rao^22^ observed an increase in sweet potato productivity under a combination of manure and mineral fertilizer in climatic conditions similar to the study area (humid tropical conditions). Besides increasing crop yields, incorporating organic inputs results in the accumulation of organic carbon that has been suggested to enhance cereal crop productivity and yield stability^23^.

A country's economic prosperity and food security rely heavily on increasing the productivity of food crops. Declining soil nutrient, particularly N, is the primary limiting nutrient for cereal crop performance across most African environments in terms of yield level and yield stability^24^. Maize is one of the main cereal food crops grown globally^25^, the predominant annual food crop in Kenya, and more so in the study area for rain-dependent smallholder farmers^14^. However, due to continuous soil inversion, low or no soil nutrient replenishment, climate variability, and unbalanced nutrient mining, yields from the small-scale fields in the study area are unstable and below 1.0 Mg ha^−1^ from a probable 6 to 8 Mg ha^−1^^5^.

Maize productivity is generally defined in terms of yield, yield stability, and attributes that interest the farmers^26^. Therefore, besides increasing crop yields, enhancing yield stability is a crucial objective of agricultural growth. Yield stability analysis aids in understanding year-to-year variability compared to the conventional reporting of average yields only^27^. In addition, stability is among the four pillars used in the definition of food security^28^. Stabilizing smallholder crop yields under varying climatic conditions requires implementing strategies focused on soil and water management in Africa^29^. Sheng-rnaol et al.^30^ reported increased maize yield stability under a combination of mineral fertilizers and farm yard manure. Furthermore, reducing soil disturbance (conservation tillage) and retaining crop residue are key strategies for soil and water conservation and sustainability of agricultural systems^31^. Stable yields denote less risk and more predictable returns, which may incentivize farmers to invest^32^ in soil and water management strategies. Greater yield stability is crucial in enhancing food security than just peak yield^33^. However, evidence of the effects of conservation strategies on crop yield and yield stability in the study area and SSA region over a medium-term period is limited. Thus, understanding the effects of conservation practices on maize yield and yield stability in the medium term is essential for sustainable agriculture. Therefore, we conducted this study to evaluate the effect of conservation management strategies (integration of conservation tillage with soil inputs) and conventional tillage (farmers' practice) on maize yield and yield stability.

## Results

### Rainfall characteristics during the study period

Rainfall is one of the most critical agro-meteorological crop production factors in the tropics, more so for rain-dependent output. We observed variations in rainfall attributes between the cropping seasons during the trial period (Table 1). The amount of rainfall varied between seasons, with short rain seasons receiving higher amounts than the long rain seasons except during LR16 and LR18. This observation agreed with Mucheru-Muna et al.^34^, who observed that rainfall amounts were higher during the short rainy seasons than in the long rain seasons in the study area. The rainfall onset and cessation dates for both the long rains and short rains seasons were within the normal range during the trial period, as reported by Ngetich et al.^35^ in the study area. Generally, the short rains season had longer growing seasons than the long rains season. The observations agreed with a study conducted in the area by Nathan et al.^3^. There were dry spells during each season, corroborating with the report of Rockström et al.^36^ and Kiboi et al.^14^.

**Table 1:** Rainfall amounts and characteristics of long and short rains seasons during the study in Meru South sub-county, Kenya.

	LR16	SR16	LR17	SR17	LR18	SR18	LR19*	SR19	LR20	SR20	LR21
Total rainfall (mm)	879	385	341	571	1159.5	590	374.6	1464.8	490	681.7	533
Onset	11th April 2016	28th Oct 2016	27th Mar 2017	20th Oct 2017	13th Mar 2018	19th Oct 2018	28th Mar 2019	5th Oct 2019	3rd Mar 2020	20th Oct 2020	18th Mar 2021
Cessation	29th Jun 2016	31st Dec 2016	30th May 2017	4th Jan 2018	31st Jul 2018	22nd Feb 2019	4 th Jun 2019	2nd Feb 2020	15th Jun 2020	22nd Feb 2021	29th Jul 2021
Length of the season	80	65	65	77	141	127	66	117	105	126	134
Dry spells											
5 to 10 days	1	3	5	2	1	1	3	3	3	1	4
11 to 15 days	1	0	0	2		1	1	0	0	1	0
More than 15 days	1	0	1	0	2	1	0	0	1	3	1

LR16, long rains 2016; SR16, short rains 2016; LR17, long rains 2017; SR17, short rains 2017; LR18, long rains 2018; SR18, short rains 2018; LR19*, long rains 2019; SR19, short rains 2019; LR20, long rains 2020; SR20, short rains 2020 and LR21, long rains 2021.

*During LR19 rainfall was poorly distributed and dry spells experienced during the vegetative and grain filling stage, thus nearly 100% crop failure was experienced in almost all treatment plots during the season therefore it was not considered during statistical analyses of the yields data.

### Grain yield

Maize grain yields significantly differed during the trial period (Table 2). Application of residue plus inorganic fertilizer plus manure (RFM) under conservation tillage had significantly higher grain yields throughout the trial than the control treatment. Yields under minimum tillage (Mt) treatment were not significantly different from the conventional tillage treatment (Control) throughout the trial period. During the LR16 season, conservation tillage with RFM, RF, RTM, and RTP significantly (*p* = 0.001) increased grain yields by 152, 118, 107, and 88%, respectively, compared to the control treatment. During SR16 and LR17 seasons, incorporating RF, RFM, and F under conservation tillage significantly increased grain yields compared with the control treatment. During the LR18 season, RFM, RF, RTP, and RML treatments significantly increased (*p* =  < 0.0001) grain yields by 160, 132, 103, and 61% compared with the control treatment (Table 2). Except for the Mt treatment, all the treatments significantly increased the yields during the SR18, LR20, and LR21 seasons compared with the control. During the SR19 season, Mt and RTP were not significantly different from the control. Generally, grain yields were significantly low during the SR16 and SR17 seasons, while there was a crop failure during the LR19 season (Table 2). Nearly 100% crop failure was experienced in almost all treatment plots during LR19, thus the season was not considered during statistical analyses. All treatments with soil inputs significantly increased grain yields compared to the control treatment during the SR18 season. Grain yields were more under the combination of fertilizer and organic inputs treatments followed by sole inorganic fertilizer use than the control. Yields from the RML treatment were not significantly different from the control treatment during the trial period's first rainy seasons (LR16–SR17) but gradually showed a significant increment later (Table 2).

**Table 2:** Maize grain yield (Mg ha^−1^) under conservation management strategies for eleven cropping seasons in Meru-South sub-county, Kenya.

Trt	LR16	SR16	LR17	SR17	LR18	SR18	LR19	SR19	LR20	SR20	LR21	%CV
Control	1.56^c^	0.14^d^	2.70^dc^	0^c^	1.39^e^	2.11^f^	0	3.10^c^	1.09^d^	0.12^d^	1.19^d^	29.3
F	1.72^c^	0.72^cb^	3.71^b^	0.36^ab^	1.8^ed^	4.57^bc^	0	6.53^a^	2.98^cb^	0.94^bc^	3.54^b^	19.2
Mt	1.61^c^	0.14^d^	1.85^d^	0^c^	1.22^e^	1.83^f^	0	3.32^c^	0.79^d^	0.36^dc^	0.95^d^	28.8
RF	3.40^ab^	1.22^a^	5.07^a^	0.29^ab^	3.32^ab^	4.80^b^	0	5.72^ab^	3.60^b^	2.00^a^	4.44^a^	14.8
RFM	3.93^a^	0.91^ab^	5.25^a^	0.45^a^	3.62^a^	5.41^a^	0	6.65^a^	4.57^a^	2.17^a^	4.75^a^	14.5
RML	2.39^cb^	0.1^d^	1.88^d^	0.01^c^	2.25^cd^	3.37^e^	0	4.66^b^	3.65^b^	0.62^bcd^	4.31^a^	17.2
RTM	3.23^ab^	0.42^cd^	3.29^bc^	0.22^abc^	1.82^ed^	3.74^de^	0	4.73^b^	3.75^ab^	1.73^a^	3.38^bc^	30.4
RTP	3.04^ab^	0.41^cd^	2.68^dc^	0.12^bc^	2.83^cb^	4.11^dc^	0	2.61^c^	2.36^c^	1.13^b^	2.77^c^	33.7
p	< 0.0001	< 0.0001	< 0.0001	0.01	< 0.0001	< 0.0001	–	< 0.0001	< 0.0001	< 0.0001	< 0.0001	

Treatment abbreviations Ct = Farmers practice, F = Inorganic fertilizer, Mt = Minimum tillage, RF = residue + inorganic fertilizer, RFM = Residue + inorganic fertilizer + Manure, RML = Residue + Manure + Legume, RTM = Residue + Tithonia + Manure, RT *p* = Residue + Tithonia + Phosphate rock.

The same superscript letters in the same column denote no significant difference between the treatment means in a season.

Honestly significant difference (HSD) at *p* = 0.05.

### Grain yield stability

**Figure 1:**
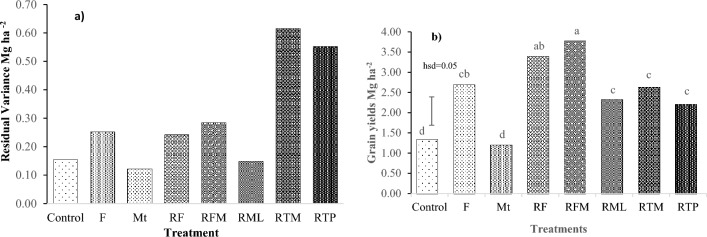
(**a**) Residual variance of grain yields in Mg ha^−2^ under conservation management strategies in Meru South sub-county over ten cropping seasons. (**b**) Average grain yields in Mg ha^−1^ under conservation management strategies in the Meru South sub-county over ten cropping seasons. Means with different letters indicate statistical differences (at *p* = 0.05) using the hsd test.

There was heterogeneity in grains yield residual variance, indicating that the treatments affected yield stability (Fig. 1a). A larger residual variance implied lesser yield stability. There were significant differences in the various factors and their interactions: season *p* < 0.0001, treatment *p* < 0.0001, and treatment × season *p* < 0.0001. The Mt treatment had the least grain yield residual variance of 0.12 Mg ha^−2^, followed by Ct and RML treatments, 0.15 Mg ha^−2^, while RTM had the highest grain residual variance of 0.62 Mg ha^−2^. This demonstrated the stability of the grain yields under Mt, Ct, and RML treatments. However, the stable treatments had lower average grain yield during the trial period (Fig. 1b). Minimum tillage (1.21 Mg ha^−2^) had the least average grain yields, followed by the control treatment (1.34 Mg ha^−2^). The RFM treatment had the highest average grain yield (3.77 Mg ha^−2^), followed by the RF treatment (3.39 Mg ha^−2^) (Fig. 1b).

## Discussion

Applying soil inputs under conservation tillage (sole inorganic fertilizer, combination of fertilizer and organics, use of sole organics, and crop residue retention) increased grain yields compared to no input use treatments, i.e., Mt and the control. This corroborated the findings of Liang et al.^37^. Reduction in tilling operations, conservation tillage with retention of crop residue, and/or inclusion of organics enhance SOC storage, thus improving soil quality and its production capacity, including crop yield^38^. Significantly high grain maize yields under the RFM treatment were attributed to improved fertilizer use efficiency due to a balanced supply of nutrients for the crop. This corroborated the findings of Jate and Lammel^39^. Increased grain yields under the sole application of organics (RTM, RTP) were attributed to quick nutrient release from Tithonia and increased soil organic carbon and matter from manure under RTM as observed and reported from same site by Kiboi et al.^14^ and^40^. *Tithonia diversifolia* is a high-quality and rapidly decomposing biomass (low C/N ratio), thus enhancing nutrient availability due to nutrient release rates^41^ under conservation tillage. The use of organic manure is widely reported to improve soil organic carbon, thus build-up of organic matter enhancing soil health and crop productivity^42^.

Low yields from the RML treatment (insignificant compared to the control) during the first rain season could be ascribed to nutrient competition between the cereal and legume, as also reported by Shisanya et al.^43^ in the study area, and delayed nutrient release due to maize stover residue retention. Maize residue is a low-quality organic material with a wide carbon-to-nitrogen ratio known to cause initial nutrient immobilization^44^. In Malawi, John et al.^45^ reported that maize yield in legume systems was generally better than continuous sole maize and was not differentiated from each other. The increase in yields later in the trial period was due to increased soil organic matter, hence increased water retention^46^. In their study, Nyirenda and Balaka^47^ found that under conservation agriculture-related practices, including intercropping maize with legumes, mulching, and reduced soil disturbance, increased biological activities on litter/residue, resulting in high production and stability in soil organic carbon and organic matter. Consequently, they observed increased maize yield under conservation agriculture compared to conventional practices. A finding was also reported by^48^. In Western Kenya, Magambo et al.^49^ observed that adopting intercropping has the highest effect on maize yield, followed closely by combining intercropping and manure.

We observed no significant difference in grain yields between the control and Mt treatments. This was attributed to no input application in the two treatments and soil compaction under conservation tillage (Mt) treatment. The findings agreed with the meta-analysis results reported by Githongo et al.^50^. Soil compaction due to reduced soil inversion under Mt treatment could have led to limited water infiltration and storage and reduced root penetration, resulting in low yield. In their meta-analysis, Rusinamhodzi et al.^10^ also reported no effect of reduced tillage under continuous maize or zero tillage on yield after ten years of experimentation.

Despite receiving rains during the SR16 and SR17 seasons, the yields were significantly low, while during the LR19 season, there was a total crop failure. The total crop failure was attributed to prolonged dry spells during the critical crop growth stages (vegetative and grain filling stage) and poor rainfall distribution resulting in soil water content deficit^14^. The rainfall amounts were so low that none of the soil management practices was able to reduce/prevent crop failure during this season. Mucheru-Muna et al.^34^ reported a similar observati on in the study area that dry spells often occurred during the peak crop water requirement periods (flowering and tussling). Too little precipitation during critical maize growth stages significantly decreases grain yield.

We attributed our results of high yield stability under conservation tillage (Mt) to increased soil organic carbon (SOC) due to reduced soil disturbance^46^. This agrees with Xu et al.^12^, who reported an increase in SOC storage under conservation tillage (subsoiling and no-tillage), enhancing yield stability. Similarly, Sileshi et al.^51^ reported that maize yield in treatments with no input was stable but had low yields. Contrary to our findings, the study by Liu et al.^52^ reported that the stability of conventional tillage was greater than that of no-tillage.

Higher maize grain stability under RML treatments could be attributed to the increased soil organic matter due to microbial biomass build-up from the *Dolichos lablab* L. legume crop and root biomass^53^. This agrees with Pan et al.^23^, who demonstrated that increased SOM aids significantly in reducing the uncertainty of annual cereal productivity, as they reported a positive correlation between SOM and cereal productivity in China. Other researchers, Seremesic et al.^54^, have also demonstrated a linear relationship between SOC storage and crop yield and yield stability. Sileshi et al.^51^ also found stable maize yields in intercropping with Leucaena (legume tree), while Mupangwa et al.^13^ observed maize yield stability in intercropping with different legumes such as common beans, soybean, and desmodium. Besides, several researchers confirm yield stability intercrop yields are more stable than sole crop yields from analysis of many experiments and studies, e.g., Rao and Willey^55^ confirmed from ninety four (94) experiments, while Raseduzzaman and Jensen^56^ analyzed thirty three (33) studies.

Generally, our results indicated that adding soil inputs and residue application under conservation tillage did not necessarily enhance yield stability but increased maize grain yield compared to the farmers' practice (control). Additionally, from the same site, Kiboi et al.^14^ reported better maize performance of critical growth factors, including chlorophyll content and plant height, under treatments with soil inputs and residue application. Similarly, Sileshi et al.^51^ also found that maize yields grown with the recommended fertilizer were unstable. Our results also corroborated with the findings of Verhulst et al.^57^, who found that conservation agriculture practices (conservation tillage with residue retention) under rain-fed conditions increased maize yield but had no significant influence on yield stability. In their meta-analysis, Rusinamhodzi et al.^10^ also reported that conservation agriculture treatment (conservation tillage with mulch) did not affect yield stability. They also outlined that the success of conservation agriculture in improving crop yields depends on appropriate targeting to climatic and edaphic conditions with adequate inputs.

In summary, the findings demonstrated enhanced maize grain yields under conservation tillage with soil inputs compared to treatments with no inputs, i.e., the Mt and control under rain-fed agricultural conditions. Yield stability analysis showed that incorporating soil inputs did not necessarily lead to stable yields. However, intercropping maize with *Dolichos lablab* L. legume and manure application positively influenced yield stability and crop yields. This highlights the potential of the forage/green manure legume in addressing productivity challenges in smallholder farming systems in the sub-humid tropical regions. The findings from the study indicated a lack of advantage of conservation tillage over conventional tillage on crop yields, as well as the use of soil inputs under conservation tillage on yield stability during the trial period. Thus, we suggest longer-term research on the effects of contrasting tillage strategies on crop yield and incorporating soil inputs in conservation tillage on yield stability under rainfed agriculture.

## Materials and methods

### Description of the study site

We conducted the study under rainfed conditions at Kangutu primary school farm (00° 98′ S, 37° 08′ E) in Meru South sub-county, Tharaka-Nithi County, Kenya^14^. Meru South sub-county is located in Upper Eastern Kenya and represents a high agricultural potential region^58^. Agriculture in the study area is characterized by small-scale mixed farming activities comprising food crops such as legumes, cereals with maize being the predominant annual food crop^14^, cash crops (e.g., coffee, tea), agroforestry including *Lantana Camara*, *Leucaena trichandra*, *Tithonia diversifolia*, and livestock such as goats, cattle^43^. The area experiences a mean temperature of 20 ℃ annually and receives a total annual rainfall of 1200 to 1400 mm. The rainfall pattern is bimodal: long rain season (LR) lasting from March to June and short rain season (SR) from late October to December. Thus, the region has two cropping seasons annually. The predominant soil type is *Humic Nitisols*, typically deep and weathered soil with moderate to high inherent fertility.

### Trial design and treatments

We designed and established the trial during the season of the long rains in 2016 (LR16). The trial was laid in a randomized complete block design (RCBD). The treatments (conservation management strategies) included conservation tillage (minimum tillage) with soil inputs and without [minimum tillage (Mt)] and conventional tillage (Control) (farmers practice)]. The soil inputs applied under conservation tillage included sole inorganic fertilizer (F), inorganic fertilizer and maize residue application (RF), inorganic fertilizer, maize residue and manure (RFM), maize residue, manure, and legume intercrop (*Dolichos Lablab* L.) (RML), maize residue, *Tithonia diversifolia* and manure (RTM) and maize residue, *Tithonia diversifolia* and phosphate rock (Minjingu) (RTP) (Table 3). Goat manure^59^ was used as the majority of farmers in the study area practice goat keeping, thus readily available. This resulted in eight treatments replicated four times. Conventional tillage (Control) was defined as the region's farmers' practice, which involved continuous land plowing and weed removal using a hand hoe without soil inputs. Most farmers in the study area do not apply soil inputs due to high input and transport costs^17^. Conservation tillage involved digging planting holes during land preparation and hand pulling of weeds from the treatment plots. Being the dominant food crop, maize (*Zea mays* L.) variety H516 was the test crop, and the treatment plot sizes measured 6 m by 4.5 m. The trial was implemented for eleven consecutive cropping seasons (Table 1).

**Table 3:** Treatments implemented at Kangutu primary school farm (00° 98′ S, 37° 08′ E).

Treatment	Abbreviation
Conventional tillage (farmers' practice)	Ct
Conservation tillage (minimum tillage)	Mt
Conservation tillage with inorganic fertilizer	F
Conservation tillage with maize residue + inorganic fertilizer	RF
Conservation tillage with maize residue + inorganic fertilizer + manure	RFM
Conservation tillage with maize residue + manure + legume intercrop (*Dolichos Lablab* L.)	RML
Conservation tillage with maize residue + *Tithonia diversifolia* L. + manure	RTM
Conservation tillage with maize residue + *Tithonia diversifolia* L. + phosphate rock (Minjingu)	RTP

### Field experiment management

Land plowing under conventional tillage (Control) was done using a hand hoe to 15 cm depth, while in conservation tillage plots, only planting holes were dug. Maize planting was done at 0.75 m in-between row spacing and 0.5 m in-row spacing, and a 1 m buffer between each plot. Three seeds were planted per hole to ensure maximum plant population. A fortnight after emergence, the extra plant was thinned out to remain with two plants per hole, thus, a population density of 53,333 plants ha^−1^. Under RML treatment, one row of maize was alternated with one row of legumes (Dolichos lablab). The spacing for legumes was 75 cm between rows by 20 cm in rows to have equal maize plant population density as in other treatments. Soil inputs applied in conservation tillage plots supplied an equivalent amount of 60 kg N ha^−1^ to meet the recommended maize nutrient requirements for the study location^60^. Phosphorus was added as Triple Super Phosphate (TSP) in the treatments with only inorganic fertilizer and treatments with the combination of inorganic fertilizer and organics at the rate of 90 kg P ha^−1^ during planting.

In the conservation tillage plots with inputs, we incorporated organics (*Tithonia diversifolia* and manure) only in the planting holes two weeks before the onset of each cropping season. We obtained *Tithonia diversifolia* from nearby biomass transfer ridges, weighed it, chopped it into small pieces, and incorporated it into the soil. Manure was obtained from the surrounding local fields, mixed thoroughly, and dried under shade for at least eight weeks. We determined the N content from a sample of each organic amendment (*Tithonia diversifolia* had 3.8% while manure had 2.1%). Afterward, the quantity of organics to be applied, equivalent to 60 or 30 kg N ha^−1^, was calculated (for the treatments with only organics, an equivalent of 60 kg N ha^−1^ was applied, and for the treatments with combination, an equivalent of 30 kg N ha^−1^ for each amendment was applied). After thinning, we uniformly applied maize residue (5 Mg ha^−1^) in five treatments under conservation tillage (Table 3). Weeding in conventional tillage plots was carried out thrice per season using a hand hoe, while under conservation tillage, it was by hand pulling when necessary. We controlled stem borers by preventive application of Tremor® GR 0.05 (a granule-formulated synthetic pyrethroid insecticide with Beta-cyfluthrin being the active ingredient) pesticide.

### Data collection

#### Rainfall received during the trial period

We recorded daily rainfall amounts using a manual rain gauge installed at about 200 m from the treatment plots.

#### Grain yield measurement

At maturity, we harvested maize grain in July to mid-August during long rain seasons and in January to February during short rain seasons, from a net plot of 21 m^2^. To minimize the edge effect, the net plot was established by leaving out the guard rows and the first and last maize plants in each row. The cobs in each plot were separated from the stover, and fresh weight was determined. The cobs were then air dried, separated from the grains through hand shelling, weighed, and the grains' moisture content determined. Grain moisture content was determined using the Dickey-John MiniGAC® moisture meter. The grain weight was corrected based on the measured moisture content, determined to 12.5% equivalence, and converted to a per-hectare basis.

### Statistical analyses

Grain yield data were subjected to analysis of variance (ANOVA) using the Mixed Procedure Model in SAS 9.4 software^61^ to obtain an F value of the effect of the model. Differences between treatment means were examined using Tukey's honestly significant difference (HSD) at *p* = 0.05. Due to the crop failure experienced during LR19, the season was not considered for statistical analyses. The residual variance was calculated using the mixed procedure in SAS 9.4 to assess grain yield stability. The data were first analyzed with a mixed model ANOVA in which the factors rainy seasons (10 levels) and treatments (8 levels) and their interactions were considered as fixed effects, while the replications were considered as the random effect (block effect). Diagnostic plots and Levene's test were performed by subjecting the absolute values of the residuals from the basic mixed model to a regular analysis of variance, which showed heterogeneity between the seasons (*p* < 0.0001) and between the treatments (*p* < 0.0001). The mixed model was then improved by specifying that the residual variance differed between the seasons or between the seasons × treatment combinations (using a 'REPEATED' statement procedure). The smallest score for Akaike's information criterion was used to select the best model^62^ to show the difference in residual variance between the treatment combinations. The mean (fixed effect) and the variance (random effect) were the two main factors used in describing the response pattern of the grain yields under the implemented treatments. Differences between factor level means were examined using Tukey's honestly significant difference at *p* = 0.05.

### Research involving plants

Compliance with the IUCN Policy Statement on Research Involving Species at Risk of Extinction and the Convention on the Trade in Endangered Species of Wild Fauna and Flora: None of the plants (Dolichos Lablab, Tithonia diversifolia, Zea mays) species used in the field experiment is endangered or at the risk of extinction.

Experimental research and field studies on Dolichos Lablab, Tithonia diversifolia, and Zea mays plants are not endangered and, hence, not subject to institutional, national, and international guidelines and legislation. However, the seeds/planting materials of Dolichos Lablab and Zea mays were purchased from an agricultural inputs stockist shop, while the cuttings of Tithonia diversifolia were from the hedges of the farms as they freely grow.

The plant collection and use was in accordance with all the relevant guidelines.

## Data Availability

The datasets generated during and/or analyzed during the study have been presented in the article.
